# Comparative Analysis of Bacterial Tick-Borne Pathogens in Questing Ticks from Sambia Peninsula, Kaliningrad Oblast, Russia: Spring and Autumn Prevalence and Public Health Risks

**DOI:** 10.3390/microorganisms13061403

**Published:** 2025-06-16

**Authors:** Alexey V. Rakov, Evgenii G. Volchev, Ketevan Petremgvdlishvili, Tatiana A. Chekanova

**Affiliations:** 1 Laboratory for Natural Focal Infections Epidemiology, Central Research Institute of Epidemiology, 111123 Moscow, Russia; ketevan0511@mail.ru (K.P.); tchekanova74@mail.ru (T.A.C.); 2 Biosafety and Radioecology NorthWest LLC, 236040 Kaliningrad, Russia; e.volchev@mail.ru

**Keywords:** *Rickettsia*, *Borrelia*, *Anaplasma*, *Ehrlichia*, ticks, *Ixodes ricinus*, *Dermacentor reticulatus*, genospecies, Kaliningrad, Russia

## Abstract

The Kaliningrad Oblast, located in the westernmost part of Russia and bordering European Union countries, is a popular tourist destination. However, limited research has been conducted on the bacteria found in ticks in this region. We, therefore, investigated the prevalence of certain bacteria, including *Borrelia*, *Rickettsia*, *Anaplasma*, and *Ehrlichia*, as well as the genospecies of the spotted fever group *Rickettsia* (SFGR) in *Ixodes ricinus* and *Dermacentor reticulatus* tick species. To accomplish this, we employed commercial qPCR for pathogen screening. We identified specific genospecies by sequencing the *gltA* and *ompA* gene fragments. In *I. ricinus* ticks, we found *Borrelia burgdorferi* sensu lato DNA in 35.6% of samples. We also found *Rickettsia helvetica* in 17.5% of ticks. Additionally, we detected *Borrelia miyamotoi* in 1.7% and *Anaplasma phagocytophilum* in 2.6%, while *Ehrlichia chaffeensis*/*Ehrlichia muris* were present in 0.6%. In *D. reticulatus* ticks, we detected only *Rickettsia conorii* subsp. *raoultii* DNA, with a prevalence of 6.1%. These findings demonstrate a substantial risk of Lyme disease and other tick-borne infections from early spring through late autumn, emphasizing the importance of ongoing monitoring for these pathogens in the region.

## 1. Introduction

Ticks are arthropods that feed on the blood of animals, including humans. They can transmit various diseases, known as tick-borne diseases (TBDs). Climate change and increased human–wildlife interactions have contributed to a rise in the number of TBD cases [1]. Millions of people are affected annually, and this number is expected to continue rising [2].

In Russia, 68 tick species have been identified across 6 genera: *Ixodes* (31 species), *Haemaphysalis* (15), *Dermacentor* (7), *Rhipicephalus* (7), *Hyalomma* (6), and *Amblyomma* (2). The latter is rarely seen in migratory bird populations [3]. The European part of Russia has been less extensively studied for tick-borne pathogen prevalence compared to the Asian part [4]. The woodland tick *Ixodes ricinus* (Linnaeus, 1758) and the meadow tick *Dermacentor reticulatus* (Fabricius, 1794) are the first and second most abundant tick species in Europe, respectively [5]. *Ixodes ricinus* is the primary vector for tick-borne encephalitis virus (TBEV) and Lyme disease [6], while *D. reticulatus* typically transmits spotted fever group rickettsiae (SFGR) such as *Rickettsia conorii* subsp. *raoultii* (basonym: *R. raoultii*) and *Rickettsia slovaca*, which cause tick-borne lymphadenopathy (TIBOLA/DEBONEL/SENLAT) [7]. Additionally, ticks can carry other bacterial pathogens such as *Borrelia miyamotoi*, *Coxiella burnetii*, and agents of human granulocytic anaplasmosis (HGA) and human monocytotropic ehrlichiosis (HME), among others.

The Kaliningrad Oblast is a semi-exclave of Russia located in Central Europe, shares borders with the European Union countries of Lithuania (north/east) and Poland (south), and has western coastlines along the Baltic Sea. The administrative center of the region is Kaliningrad, formerly known as Königsberg. The Kaliningrad Oblast is famous for its resort towns, including those located on the Sambia Peninsula, such as Zelenogradsk, Svetlogorsk, Yantarny, and Pionersky. These towns were the focus of our study.

However, data on pathogens in *I. ricinus* and *D. reticulatus* ticks in the Kaliningrad Oblast is still limited. A recent study of *I. ricinus* ticks from natural biotopes in the Kaliningrad Oblast, based on *p66* gene sequencing, revealed the presence of four different genospecies from the *Borrelia burgdorferi* sensu lato (s.l.) complex: *Borrelia afzelii*, *Borrelia garinii*, *Borrelia valaisiana*, and *Borrelia lusitaniae*. The expected presence of *Rickettsia helvetica* was found in woodland ticks, while *R. raoultii* was detected in meadow ticks [4], aligning with patterns in neighboring Poland [8], Lithuania [9], and Belarus [10]. To better assess the prevalence and distribution of tick-borne pathogens in the Kaliningrad Oblast, further studies are needed.

According to the public data from the 2023 state report by Rospotrebnadzor, the number of people bitten by ticks increased by 18.3% compared to the previous year (6366 cases) [11]. As part of epizootic surveillance, 737 field-collected *I. ricinus* ticks were tested for *Borrelia* spp. using PCR, and *Borrelia* spp. were detected in 83 samples (11.26%). In total, 6653 ticks removed from humans were examined, and *Borrelia* spp. were identified in 910 cases (15.06%). Other bacterial TBD agents were not tested [11].

TBD epidemiology reflects both the geographic distribution and seasonal activity of vectors and their hosts involved in pathogen transmission [12,13]. In the Kaliningrad Oblast, the activity of *I. ricinus* and *D. reticulatus* remains high throughout the summer and autumn, with peak activity observed from late April to early May [11].

TBDs are expanding globally due to climate change and human-induced environmental modifications. Therefore, it is essential to continuously monitor both natural and urban environments. Studying the potential vectors and reservoirs of TBD pathogens should be integrated into routine environmental monitoring programs. Healthcare professionals should be promptly informed about the spread of ticks and associated pathogens in their region, allowing for timely diagnosis and prevention measures.

The aim of this study was to compare the prevalence of major bacterial tick-borne pathogens, belonging to the genera *Borrelia*, *Rickettsia*, *Coxiella*, *Anaplasma*, and *Ehrlichia*, in questing ticks collected from vegetation on the Sambia Peninsula (the Kaliningrad Oblast) during the spring and autumn peak activity periods.

## 2. Materials and Methods

### 2.1. Study Area

Climate: The climate of the Kaliningrad Oblast is transitional, shifting from temperate continental to maritime. On average, rain falls for 185 days a year, snow for 55 days, and there are about 60 cloudy days and 68 sunny days. The summers and frosts are short, and the snow cover does not last long. The mean air temperature in the region is approximately +8 °C [14].

Flora and fauna: The region’s topography consists of hilly plains. The vegetation is predominantly meadowland, with a small proportion of artificially established mixed forests. Approximately one-third of the land area comprises hayfields and pasture meadows, supporting around 30 herbaceous plant species [15]. The area hosts diverse fauna and lies along the ancient migratory route of birds traversing the Curonian and Baltic Spits from northern Europe to southern Europe and North Africa.

### 2.2. Tick Collection

**Figure 1:**
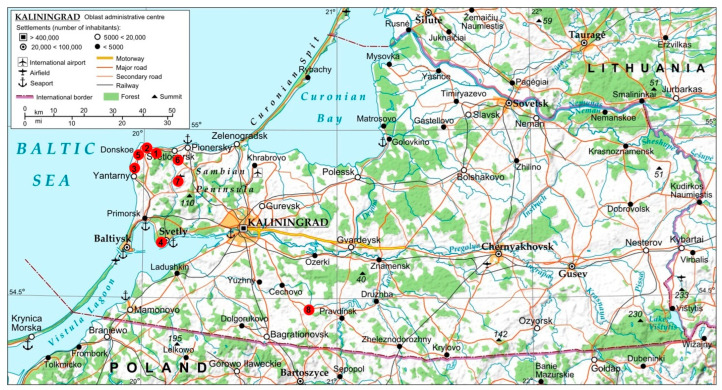
Map of tick collection sites in the Kaliningrad Oblast. The collection sites are marked with red circles (details are provided in Supplementary Table S1): 1—Primorye (Groß Kuhren); 2—Filinskaya Bukhta (Klein Kuhren); 3—Sinyavinskoye Ozero (Groß Hubnicken); 4—Baltiyskiy Les, Svetly (Zimmerbude); 5—Donskoye (Groß Dirschkeim); 6—Salskoe (Sankt Lorenz); 7—Dunaevka (Lopsienen); 8—Filippovka (Dommelkeim). Modified from https://commons.wikimedia.org/wiki/File:KALININGRAD_FINAL.svg; accessed on 6 June 2025.

The tick collection sites (including collection dates, coordinates, and the name of the nearby settlement) are detailed in Figure 1 and Supplementary Table S1. Most ticks were collected across four districts of the Sambia Peninsula: Svetlogorsk, Zelenogradsk, Yantarny, and Svetlovsky. Additionally, a control group of 10 ticks (2%) was sampled in Pravdinsky District, located 12 km from the Polish border.

A total of 508 free-living questing ticks of two species (*I. ricinus* and *D. reticulatus*) were examined. These included 293 ticks collected during May–June 2023 and 215 ticks in September 2023. Among these, 343 ticks belonged to *I. ricinus* and 165 to *D. reticulatus*. The ticks were morphologically identified using the standard taxonomic keys [16]. They were collected from vegetation using a flagging method. Briefly, ticks were collected during daylight hours by dragging a 1.5 × 2.0 m flag over vegetation in the specified study zones. All the ticks were starved (unfed). Ticks attached to the flag were removed with tweezers, placed into individual Eppendorf tubes, and stored at −70 °C until they were transported to the laboratory. Transportation to the laboratory was carried out by air in a thermally insulated container with ice packs within 3 days. Homogenization and DNA extraction were performed within a week of arrival at the laboratory. The extracted DNA and the remains of the homogenates were stored at −20 °C for one month, when PCR and sequencing were performed. Subsequently, all the nucleic acid residues and homogenates were transferred to −70 °C for long-term preservation.

### 2.3. DNA Extraction and Quantitative PCR

Each tick was individually washed with 96% ethanol and then 0.15 M sodium chloride solution. Ticks were homogenized in a 2.0 mL Eppendorf tube containing 300 µL of 0.15 M sodium chloride with tungsten carbide beads in a TissueLyser LT homogenizer (Qiagen, Hilden, Germany) at 50 Hz for 10 min. The total DNA was extracted from 100 µL of supernatant using the “AmpliSens^®^ RIBO-Prep” kit (CRIE, Moscow, Russia). qPCR screening was performed for SFGR using the “AmpliSens^®^  *Rickettsia* spp. SFG-FL” kit targeting the *ompB* gene, for *C. burnetii* using the “AmpliSens^®^  *Coxiella burnetii*-FL” kit, for *B. miyamotoi* using the “AmpliSens^®^  *Borrelia miyamotoi*-FL” kit targeting the *glpQ* gene, for TBEV, *B. burgdorferi* s.l., *A. phagocytophilum*, *E. chaffeensis*/*E. muris* using the “AmpliSens^®^ TBEV, *B. burgdorferi* sl, *A. phagocytophilum*, *E. chaffeensis*/*E. muris*-FL” kit according to the manufacturer’s instructions. All the kits are manufactured by CRIE, Moscow, Russia. The Rotor-Gene Q qPCR thermocycler (Qiagen, Hilden, Germany) was used.

### 2.4. Conventional PCR and Sanger Sequencing

The genospecies of SFGR was determined by Sanger sequencing of the citrate synthase *gltA* (384 bp) and outer membrane protein A *ompA* (532 bp) partial genes using both DNA strands with specific primers, as described previously [17]. Homologous sequences were identified in the GenBank nr/nt database using BLASTN 2.16.0 with the default parameters.

### 2.5. Phylogenetic and Statistical Analysis

Dendrograms were constructed using MEGA 6.06 software with the maximum likelihood method on aligned sequences of both genes, with 1000 bootstrap replicates. For comparison, homologous sequences of the complete genomes of representative SFG rickettsiae from GenBank were used. A homologous fragment of the *Rickettsia bellii* An04 genome sequence (NZ_CP015010) was used as an outgroup to construct a dendrogram using the *gltA* partial gene sequence.

For the tick infection rates, 95% confidence intervals (CIs) were calculated using a modified Wald method in QuickCalcs (GraphPad, San Diego, CA, USA). The two-sample *z*-test to compare sample proportions was performed using Epitools [18]. A *p*-value ≤ 0.05 was considered statistically significant.

The sequences from this study are available in GenBank (Acc. No. PV520512-PV520536).

## 3. Results

The qPCR screening of 508 tick samples revealed the presence of DNA from 5 pathogens in *I. ricinus* (SFGR, *B. burgdorferi* s.l., *B. miyamotoi*, *A. phagocytophilum*, *E. chaffeensis*/*E. muris*) and only SFGR in *D. reticulatus* (Table 1). The most frequently detected pathogens in *I. ricinus* were those belonging to the *B. burgdorferi* s.l. complex (35.6%, 95% CI 30.7–40.8%). SFGR were the second most common (17.5%, 95% CI 13.8–21.9%). The remaining pathogens were found significantly less often, in 0.6–2.6% of all tested *I. ricinus* ticks. In *D. reticulatus*, only 6.1% (95% CI 3.2–10.9%) of tested samples were positive for SFGR. Among the *D. reticulatus* samples examined, no genetic material of *Borrelia*, *Anaplasma*, or *Ehrlichia* was detected. Additionally, the DNA of *C. burnetii* and TBEV was not found in any of the ticks of either species.

**Figure 2:**
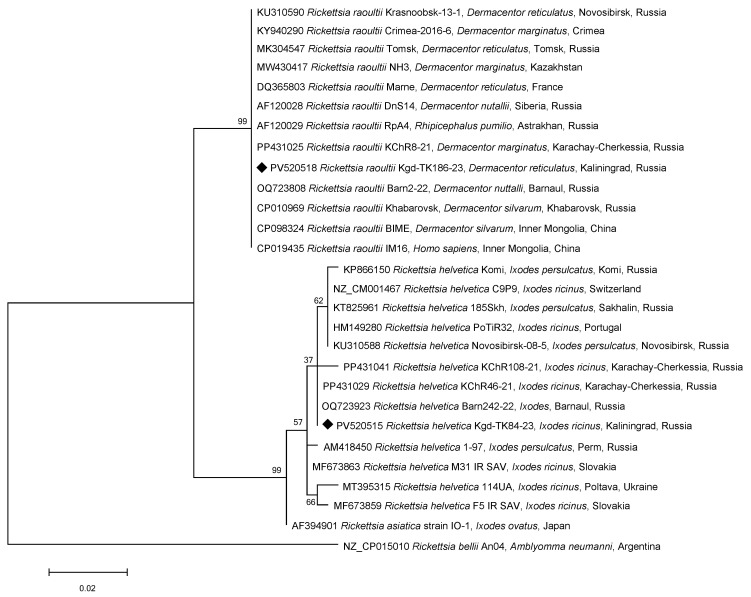
Phylogenetic tree constructed using the maximum likelihood method based on nucleotide sequences of *Rickettsia* spp. from ticks, including ones from this study (Kaliningrad, black diamonds, ♦) and reference sequences of the *gltA* gene fragment (384 bp). The *R. bellii* An04 (NZ_CP015010) sequence was used as an outgroup. The GenBank accession numbers for reference sequences are shown with the sequence name, tick species, and country. The branch numbers indicate bootstrap support (1000 replicates). The scale bar indicates phylogenetic distance.

**Figure 3:**
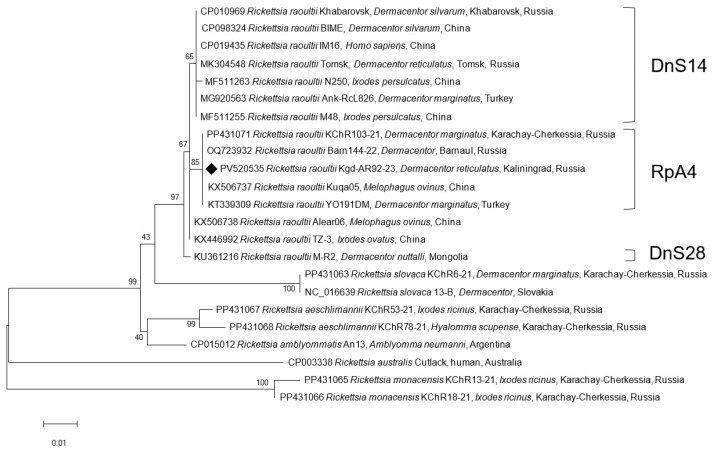
Phylogenetic tree constructed using the maximum likelihood method based on nucleotide sequences of *Rickettsia* spp. from ticks, including one from this study (Kaliningrad, black diamonds, ♦) and reference sequences of the *ompA* gene fragment (532 bp). Genotype clusters DnS14, RpA4, and DnS28 are presented for *R. raoultii*. The GenBank accession numbers for reference sequences are shown with the sequence name, tick species, and country. The branch numbers indicate bootstrap support (1000 replicates). The scale bar indicates phylogenetic distance.

To assess the SFGR genospecies, 13 of 60 randomly selected ticks of *I. ricinus* (1–2 ticks per site/season) and all 10 *D. reticulatus* (100%) positive for *Rickettsia* spp. were tested. Since there was a possibility that, in addition to *R. raoultii*, DNA from another *Rickettsia* species, namely *R. slovaca*, could be present in this tick species, the *gltA* gene fragment was sequenced in all 10 SFGR-positive samples (Table 2). All the sequences were 100% identical to all the reference *R. raoultii* genomes (Figure 2). For genotype determination, we sequenced a partial *ompA* gene in two randomly selected samples from the 10 *R. raoultii*-positive specimens. These sequences were 100% identical to our previous findings from Barnaul and Karachay-Cherkessia [17,19] and were classified as the RpA4 genotype, forming a distinct phylogenetic cluster (Figure 3). The reference *R. raoultii* strains Khabarovsk, IM16, and BIME clustered separately as the DnS14 genotype [20]. This cluster also included samples from ticks collected in Tomsk (Russia) and China. The DnS28 genotype is represented on the tree by the strain M-R2 from Mongolian *Dermacentor nuttalli*.

Similarly, *R. helvetica* DNA was detected in 13 out of 60 SFGR-positive *I. ricinus* tick samples by sequencing the partial *gltA* gene (Table 2). These findings suggest that the remaining 47 untested samples also contain *R. helvetica* DNA. Since *R. helvetica* lacks the *ompA* gene, only the phylogenetic relationships based on the *gltA* partial gene could be assessed. Notably, sequences from ticks collected in Barnaul and Karachay-Cherkessia [17,19] were 100% identical and differed from sequences from Novosibirsk, Sakhalin, and Komi by 1–2 synonymous nucleotide substitutions (Figure 2). In the control group, collected in the Pradinskoye District, *I. ricinus* ticks contained one sample each of *R. helvetica* and *B. burgdorferi* s.l. (Table 3).

**Figure 4:**
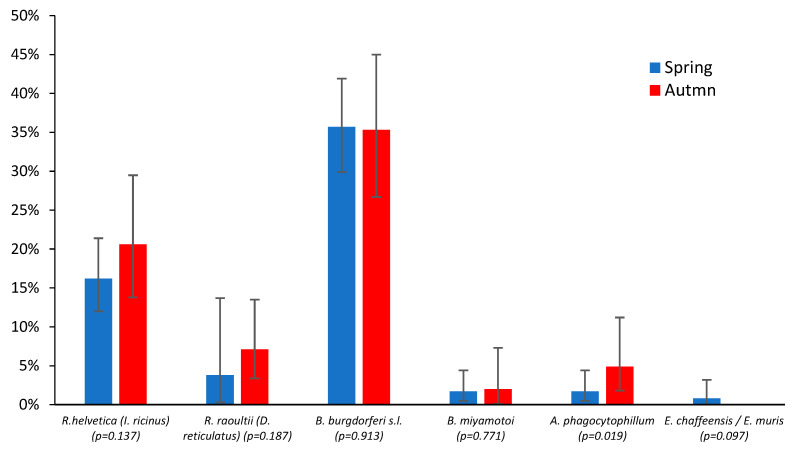
Comparison of tick infection rates for *I. ricinus* and *D. reticulatus* with bacterial pathogens during the spring (blue) and autumn (red) periods of peak tick activity (as a percentage of the total examined ticks). The p-value level of significance is shown in parentheses.

Tick activity in Central Europe typically exhibits bimodal seasonality, with peak abundance occurring during the spring (March–June) and autumn (October–November) periods, and reduced activity during the midsummer (July) [21]. To assess seasonal variation in pathogen prevalence, we compared infection rates between spring (Table 3) and autumn (Table 4) peak activity periods. The infection rates for *Rickettsia* spp. across both tick species and *Borrelia* spp. in *I. ricinus* showed comparable prevalence levels (Figure 4). All the minor observed differences in pathogen prevalence were statistically insignificant (*p* > 0.05), except for *A. phagocytophilum*, which can be explained by its low detection frequency: four cases in the spring peak vs. five in the autumn.

Co-infections with double, and in one case, triple, pathogens were registered in 25 (7.3%) of the studied *I. ricinus* ticks (Table 5). In one case, the DNA of *R. helvetica*, *B. miyamotoi*, and *B. burgdorferi* s.l. was detected in a single tick. The most frequent co-infection was *R. helvetica* + *B. burgdorferi* s.l. (4.7% of all ticks). Other combinations were observed in one to three ticks each (Table 5). No significant difference was observed in the co-infection rates between the spring (7.5%) and autumn (6.9%) activity peaks (*p* = 0.84). No co-infections were detected in *D. reticulatus*, as only *R. raoultii* was present in this species.

## 4. Discussion

Field-based monitoring of TBD pathogens plays a crucial role in epidemiological surveillance. This information helps healthcare professionals understand infection rates and temporal dynamics, enabling risk assessment and the timely implementation of preventive interventions.

In this study, we present the first comprehensive analysis of bacterial tick-borne pathogen prevalence in ticks from the westernmost part of the Sambia Peninsula. A high infection rate of *D. reticulatus* ticks with *R. raoultii* was detected (17.5%, 95% CI 13.8–21.9%), nearly three times higher than that of *I. ricinus* ticks infected with *R. helvetica* (6.1%, 95% CI 3.2–10.9%). Additionally, *I. ricinus* ticks harbored DNA from multiple pathogens: *B. burgdorferi* s.l. (35.6%, 95% CI 30.7–40.8%), *B. miyamotoi* (1.7%, 95% CI 0.7–3.9%), *A. phagocytophilum* (2.6%, 95% CI 1.3–5.0%), and *E. chaffeensis*/*E. muris* (0.6%, 95% CI 0.02–2.2%).

The infection rate of *I. ricinus* with the *B. burgdorferi* s.l. complex was 35.6% (122/343, 95% CI 30.7–40.8%), which is statistically significantly more than twice as high as in another study of this tick species in the Kaliningrad Oblast—15.5% (28/862, 95% CI 13.2–18.1%) (*p* < 0.0001) [4] and three times higher according to the data of the state report “On the Status of Sanitary and Epidemiological Surveillance of the Population in the Kaliningrad Oblast in 2023” (11.26%) (*p* = 0) [11]. These observed prevalence differences, as well as variations in infection rates with other pathogens, may be explained by methodological discrepancies, specifically, the use of highly sensitive qPCR with a commercial kit in our study compared to the less sensitive conventional PCR without nested amplification employed in the other study [4]. Moreover, our sampling sites were strategically selected in high-traffic areas frequented by both residents and tourists, primarily along the Baltic Sea coast, while the sampling sites from the previous study were not as carefully chosen (Figure 1).

In neighboring Lithuania, the average *B. burgdorferi* s.l. prevalence in *I. ricinus* was 13.4%, with regional variations ranging from 1% to 35% [22,23,24,25]. In neighboring Poland, *I. ricinus* infection rates varied across studies: 11.8% [26], 14.0% [27], 20.2% [28], 26.4% [29], and 33.6% [30]. We detected *B. miyamotoi* DNA in 1.7% (95% CI 0.7–3.9%) of ticks, which is in line with previously reported rates in Poland: 0.94% [29], 2.0% [27], and 2.5% [30].

The overall SFGR infection rate in both tick species was 13.8% (70/508, 95% CI 11.0–17.1%), consistent with data from another study reporting 11.5% (191/1665; 95% CI 10.2–13.1%) [4]. However, among *I. ricinus* ticks, *Rickettsia* DNA was found in 60 out of 343 samples (17.5%, 95% CI 13.8–21.9%), which is statistically significantly higher than the 2.6% (22/862, 95% CI 1.7–3.8%) reported previously (*p* = 0). In contrast, rickettsial DNA was detected in 10 out of 165 *D. reticulatus* ticks (6.1%, 95% CI 3.2–10.9%), markedly lower than the 21.1% (169/803; 95% CI 18.4–24.0%) reported elsewhere (*p* = 0.001) [4].

Our data better corresponds with findings from Lithuania and Poland. Lithuanian studies reported a *Rickettsia* spp. prevalence of 17% in *I. ricinus* and 4.9% in *D. reticulatus* populations [9]. In Poland, studies demonstrated wider variability. *Rickettsia helvetica* DNA was detected in 3.3% [8], 3.69% [31], 7.9% [32], 10.6% [29], 10.7% [33], and 27.5% [34] of *I. ricinus* samples. However, *R. raoultii* demonstrated substantially higher prevalence in *D. reticulatus* populations, ranging from 27.1% [35], 30.7–37.7% [36], 37.8% [29], 40.7% [37], 42.8% [34], 44.1% [38], 53.0% [39] to 56.7% [8] and up to 60.9% [40]. Notably, *R. raoultii* in Poland, like in our samples, belongs to the RpA4 genotype, which predominates in European populations [37].

It is known that other genospecies of SFGR are also found in *I. ricinus* and *D. reticulatus* ticks in neighboring countries. For example, in Poland, *Rickettsia monacensis* has occasionally been detected in *I. ricinus* alongside *R. helvetica*, with prevalence ranging from 0.3% [32] to 10% [40]. Similarly, *D. reticulatus* ticks in Poland have been found to carry *R. slovaca* in 2.1% of cases [8], in addition to *R. raoultii*. Moreover, *R. monacensis* has been reported in Belarus [10]. In Russia, these genospecies have also been detected in other regions [19].

Using qPCR, we detected *A. phagocytophilum* DNA in 2.6% (95% CI 1.3–5.0%) of the tick samples, which is slightly higher than the 1.4% (95% CI 0.8–2.5%) reported in another study using conventional non-nested PCR on *I. ricinus* ticks [4]. Interestingly, in *I. ricinus* ticks collected from the Curonian Spit in 2006–2008, the prevalence of *A. phagocytophilum* DNA by qPCR was 13.4% of all the examined ticks [41]. In Poland, the prevalence of *A. phagocytophilum* in *I. ricinus* was reported as 0.3% [26], 0.54% [31], and 1.7% [42].

The infection rate of *I. ricinus* ticks with *Ehrlichia* spp., including *E. chaffeensis* and *E. muris*, was 0.6% (95% CI 0.02–2.2%). In *I. ricinus* ticks collected in 1997–1998 from the Baltic coast near Saint Petersburg and the Curonian Spit, the percentage of *Ehrlichia* spp.-positive ticks was 8.6%, of which 0.6% belonged to the former *Ehrlichia phagocytophila* complex, now classified as *A. phagocytophilum* [43]. A lower prevalence of 0.3% *Ehrlichia* spp. was reported in Polish *I. ricinus* populations [42].

All the bacteria studied are human tick-borne pathogens that have been identified. Both *Rickettsia* genospecies detected in the SFGR are pathogenic and can cause mild rickettsioses. *Rickettsia raoultii* is responsible for a disease called TIBOLA, also known as DEBONEL or SENLAT, which is characterized by a scalp eschar and cervical lymphadenopathy [44]. *Rickettsia helvetica* may cause infections with symptoms such as headache, an occasional rash, and inoculation eschar [12]. *Borrelia burgdorferi* s.l. can cause Lyme disease, while *B. miyamotoi* is responsible for hard tick relapsing fever (HTRF), also known as *B. miyamotoi* disease (BMD) [45]. *Anaplasma phagocytophilum* and *E. chaffeensis*/*E. muris* are known etiological agents of human granulocytic anaplasmosis (HGA) and human monocytotropic ehrlichiosis (HME), respectively [46,47]. *Ixodes* ticks (particularly *I. ricinus*) serve as the main vectors for these pathogens.

The relatively frequent co-infection of SFGR and *B. burgdorferi* s.l. that we detected in *I. ricinus* (4.7%, 95% CI 2.4–6.9%) has also been reported in other countries. For instance, in Poland, which borders the Kaliningrad Oblast, co-infection in *I. ricinus* was found in 4.25% [30], 4.8% [42], and 5.0% [48] of cases, which aligns well with our data. It is believed that the high frequency of SFGR + *Borrelia* co-infection is due to the fact that they do not compete with each other. Each pathogen occupies different niches within the tick: *Rickettsia* spp. mainly colonize salivary glands and ovaries, while *Borrelia* spp. bacteria predominantly inhabit midgut cells [49].

Few studies have been conducted on this subject in the Kaliningrad Oblast in the past, and the results of these studies are inconclusive. A study conducted in 2008 found that ticks collected from migratory birds at the Rybachy Biological Station on the Curonian Spit contained three *Rickettsia* species: *R. helvetica* (10.3%), *R. monacensis* (3.9%), and *Rickettsia japonica* (0.8%) [50]. A follow-up study in 2009 also detected the DNA of *B. burgdorferi* s.l. (5.9%), *R. helvetica* (11.8%), and *A. phagocytophilum* (1.5%) in *I. ricinus* ticks, as well as one case of *Rickettsia aeschlimannii* in a *Hyalomma marginatum* tick [51]. Both studies focused exclusively on migratory bird populations, so their findings may not be directly applicable to the local infection patterns in other regions.

Our study reveals a high prevalence of *B. burgdorferi* s.l. (35.6%) and SFGR (17.5% in *I. ricinus*, 6.1% in *D. reticulatus*) in ticks from the Sambia Peninsula, which significantly exceeds previous estimates for the Kaliningrad Oblast. The discrepancy between the detected prevalence and clinical reports suggests a possible underdiagnosis of TBDs in the region. The data we obtained emphasizes the critical need for continuous monitoring of bacterial tick-borne pathogens in natural foci of the Kaliningrad Oblast. We also need to further improve diagnostic methods and preventive measures. This includes better detection of human cases of Lyme disease, rickettsioses, and other TBDs, such as BMD, HME, and HGA. It is also important to consider co-infections when developing strategies for prevention and treatment.

## Figures and Tables

**Table 1:** Prevalence of bacterial tick-borne pathogens in ticks from the Kaliningrad Oblast, 2023.

Tick Species	Number of Ticks	Number of Ticks Infected by (%, 95% CI)					
		*Rickettsia* spp. SFG	*B. burgdorferi* s.l.	*B. miyamotoi*	*C. burnetii*	*A. phagocytophillum*	*E. chaffeensis/E. muris*
*I. ricinus*	343	60 (17.5%, 13.8–21.9%)	122 (35.6%, 30.7–40.8%)	6 (1.7%, 0.7–3.9%)	0	9 (2.6%, 1.3–5.0%)	2 (0.6%, 0.02–2.2%)
*D. reticulatus*	165	10 (6.1%, 3.2–10.9%)	0	0	0	0	0
Total	508	70 (13.8%, 11.0–17.1%)	122 (24.0%, 20.5–27.9%)	6 (1.2%, 0.5–2.6%)	0	9 (1.8%, 0.9–3.4%)	2 (0.4%, 0.01–1.5%)

**Table 2:** Prevalence of tick-borne rickettsioses pathogens in ticks from the Kaliningrad Oblast, 2023.

Tick Species	Number of Ticks	Number of Ticks Infected by SFGR (%, 95% CI)			
		*R. raoultii*	*R. helvetica*	Not Sequenced	Total *Rickettsia* spp.
*I. ricinus*	343	0	13 (3.8%, 2.2–6.4%)	47 (13.7%, 10.4–17.8%)	60 (17.5%, 13.8–21.9%)
*D. reticulatus*	165	10 (6.1%, 3.2–10.9%)	0	0	10 (6.1%, 3.2–10.9%)
Total	508	10 (2.0%, 1.0–3.6%)	13 (2.6%, 1.5–4.4%)	47 (9.2%, 7.0–12.1%)	70 (13.8%, 11.0–17.1%)

**Table 3:** Prevalence of bacterial tick-borne pathogens in ticks from the Kaliningrad Oblast, May–June 2023.

Tick Species	Collection Zone	Number of Ticks	Number of Ticks Infected by (%, 95% CI)				
			*Rickettsia* spp. SFG	*B. burgdorferi* s.l.	*B. miyamotoi*	*A. phagocytophillum*	*E. chaffeensis/E. muris*
*I. ricinus*	1 *	20	1 (5.0%, 0–25.4%)	4 (20.0%, 7.5–42.2%)	0	1 (5.0%, 0–25.4%)	0
	2 *	23	6 (26.1%, 12.3–46.8%)	9 (39.1%, 22.1–59.3%)	2 (8.7%, 1.2–28.0%)	0	0
	3 *	110	18 (16.4%, 10.5–24.5%)	44 (40.0%, 31.3–49.3%)	2 (1.8%, 0.1–6.8%)	0	0
	4 *	7	2 (28.6%, 7.6–64.8%)	3 (42.9%, 15.7–75.0%)	0	0	0
	5 *	78	11 (14.1%, 7.9–23.7%)	25 (32.0%, 22.7–43.1%)	0	3 (3.8%, 0.9–11.2%)	2 (2.6%, 0.2–9.4%)
	6 *	3	1 (33.3%, 5.6–79.8%)	1 (33.3%, 5.6–79.8%)	0	0	0
	Subtotal	241	39 (16.2%, 12.0–21.4%)	86 (35.7%, 29.9–41.9%)	4 (1.7%, 0.5–4.4%)	4 (1.7%, 0.5–4.4%)	2 (0.8%, 0.03–3.2%)
*D. reticulatus*	1 *	4	0	0	0	0	0
	2 *	20	0	0	0	0	0
	3 *	20	2 (10.0%, 15.7–31.3%)	0	0	0	0
	4 *	-	-	-	-	-	-
	5 *	1	0	0	0	0	0
	6 *	7	0	0	0	0	0
	Subtotal	52	2 (3.8%, 0.3–13.7%)	0	0	0	0
Total		293	41 (14.0%, 10.5–18.5%)	86 (29.3%, 24.4–34.8%)	4 (1.4%, 0.4–3.7%)	4 (1.4%, 0.4–3.7%)	2 (0.7%, 0.02–2.6%)

* 1—Primorye (Groß Kuhren), Svetlogorsk District (54.937289°, 20.051557°), collection date: 26 May 2023; 2—Filinskaya Bukhta (Klein Kuhren), Svetlogorsk District (54.942472°, 20.021023°), collection date: 21 May 2023; 3—Sinyavinskoye Ozero (Groß Hubnicken), Yantarny District (54.891063°, 19.962137°), collection date: 17 June 2023; 4—Donskoye (Groß Dirschkeim), Svetlogorsk District (54.937928°, 19.962673°), collection date: 18 June 2023; 5—Baltiyskiy Les, Svetly (Zimmerbude), Svetlovsky District (54.714097°, 19.942975°), collection date: 22 June 2023; 6—Filippovka (Dommelkeim), Pravdinsky District (54.474473°, 20.841418°), collection date: 22 May 2023.

**Table 4:** Prevalence of bacterial tick-borne pathogens in ticks from the Kaliningrad Oblast, July and September 2023.

Tick Species	Collection Zone	Number of Ticks	Number of Ticks Infected by (%, 95% CI)				
			*Rickettsia* spp. SFG	*B. burgdorferi* s.l.	*B. miyamotoi*	*A. phagocytophillum*	*E. chaffeensis/E. muris*
*I. ricinus*	1 *	3	0	0	0	0	0
	2 *	1	0	0	0	0	0
	3 *	22	3 (13.6%, 3.9–34.2%)	6 (27.3%, 12.9–48.4%)	1 (4.5%, 0–23.5%)	0	0
	4 *	4	0	3 (75.0%, 28.9–96.6%)	0	0	0
	5 *	70	18 (25.7%, 16.8–37.1%)	26 (37.1%, 26.7–48.9%)	1 (1.4%, 0–8.4%)	5 (7.1%, 2.7–16.0%)	0
	6 *	2	0	1 (50.0%, 9.4–90.5%)	0	0	0
	Subtotal	102	21 (20.6%, 13.8–29.5%)	36 (35.3%, 26.7–45.0%)	2 (2.0%, 0.1–7.3%)	5 (4.9%, 1.8–11.2%)	0
*D. reticulatus*	1 *	-	-	-	-	-	-
	2 *	44	7 (15.9%, 7.6–29.7%)	0	0	0	0
	3 *	38	0	0	0	0	0
	4 *	-	-	-	-	-	-
	5 *	31	1 (3.2%, 0–17.6%)	0	0	0	0
	6 *	-	-	-	-	-	-
	Subtotal	113	8 (7.1%, 3.4–13.5%)	0	0	0	0
Total		215	29 (13.5%, 9.5–18.7%)	36 (16.7%, 12.3–22.3%)	2 (1.0%, 0–3.5%)	5 (2.3%, 0.8–5.5%)	0

* 1—Primorye (Groß Kuhren), Svetlogorsk District (54.937709°, 20.043021°), collection date: 27 September 2023; 2—Filinskaya Bukhta (Klein Kuhren), Svetlogorsk District (54.944593°, 20.023484°), collection date: 27 September 2023; 3—Sinyavinskoye Ozero (Groß Hubnicken), Yantarny District (54.891063°, 19.962137°), collection date: 5 September 2023; 4—Salskoye (Sankt Lorenz), Zelenogradsky District (54.917200°, 20.173978°), collection date: 28 July 2023; 5—Baltiyskiy Les, Svetly (Zimmerbude), Svetlovsky District (54.714097°, 19.942975°), collection date: 3 September 2023; 6—Dunayevka (Lopsienen), Zelenogradsky District (54.867611°, 20.165678°), collection date: 3 September 2023.

**Table 5:** Co-infection of different bacterial tick-borne pathogens in the Kaliningrad Oblast, 2023.

Pathogen Co-Infections	No. of Positive	Positive Rate (95% CI)
*R. helvetica* + *B. miyamotoi* + *B. burgdorferi* s.l.	1	0.3% (0.28–0.86%)
*R. helvetica* + *B. burgdorferi* s.l.	2	0.6% (0.22–1.4%)
*R. helvetica* + *B. miyamotoi*	1	0.3% (0.28–0.86%)
*R. helvetica* + *E. chaffeensis*/*E. muris*	1	0.3% (0.28–0.86%)
*R.* spp. + *B. burgdorferi* s.l.	14	4.1% (2.0–6.2%)
*R.* spp. + *A. phagocytophilum*	1	0.3% (0.28–0.86%)
*B. burgdorferi* s.l. + *A. phagocytophilum*	2	0.6% (0.2–1.4%)
*B. burgdorferi* s.l. + *B. miyamotoi*	3	0.9% (0.1–1.9%)
Total	25	7.3% (4.6–10.0%)

## Data Availability

The sequences from this study are available in the NCBI GenBank under accession numbers PV520512–PV520536.

## Supplementary Materials

The following supporting information can be downloaded at https://www.mdpi.com/article/10.3390/microorganisms13061403/s1, Table S1: The tick collection sites in the Kaliningrad Oblast, 2023.

## Abbreviations

The following abbreviations are used in this manuscript:

TBD	Tick-borne disease
TBEV	Tick-borne encephalitis virus
SFGR	Spotted fever group *Rickettsia*
TIBOLA	Tick-borne lymphadenopathy
DEBONEL	*Dermacentor*-borne necrosis erythema and lymphadenopathy
SENLAT	Scalp eschar and neck lymphadenopathy after tick bite
HGA	Human granulocytic anaplasmosis
HME	Human monocytotropic ehrlichiosis
PCR	Polymerase chain reaction
qPCR	Quantitative polymerase chain reaction
CRIE	Central Research Institute of Epidemiology
CI	Confidence intervals
HTRF	Hard tick relapsing fever
BMD	*Borrelia miyamotoi* disease
