# Supplementary material for: Comparative Analysis of Bacterial Tick-Borne Pathogens in Questing Ticks from Sambia Peninsula, Kaliningrad Oblast, Russia: Spring and Autumn Prevalence and Public Health Risks
Source: Microorganisms. 2025 Jun 16;13(6):1403. doi: 10.3390/microorganisms13061403 (PMC12195733; doi:10.3390/microorganisms13061403)
Supplement: Supplementary file 1 [file microorganisms-13-01403-s001.zip › microorganisms-3667295-supplementary.pdf]

Table S1. The tick collection sites in the Kaliningrad Oblast, 2023.

Collection Site Name (German Historical Toponym Prior to 1946)	Urban District	Geographic Coordinates	Collection Date	Tick Species	Number of Ticks
Primorye (Groß Kuhren)	Svetlogorsky District	54.937709°, 20.043021°	26.05.2023	*I. ricinus*	20
				D. reticulatus	4
			27.09.2023	*I. ricinus*	3
Filinskaya Bukhta (Klein Kuhren)	Svetlogorsky District	54.944593°, 20.023484°	21.05.2023	*I. ricinus*	23
				D. reticulatus	20
			27.09.2023	*I. ricinus*	1
				D. reticulatus	44
Sinyavinskoye Ozero (Groß Hubnicken)	Yantarny District	54.891063°, 19.962137°	17.06.2023	*I. ricinus*	110
				D. reticulatus	20
			05.09.2023	*I. ricinus*	22
				D. reticulatus	38
Baltiyskiy Les, Svetly (Zimmerbude)	Svetlovsky District	54.714097°, 19.942975°	22.06.2023	*I. ricinus*	78
				D. reticulatus	1
			03.09.2023	*I. ricinus*	70
				D. reticulatus	31
Donskoye (Groß Dirschkeim)	Svetlogorsky District	54.937928°, 19.962673°	18.06.2023	*I. ricinus*	7
Salskoe (Sankt Lorenz)	Zelenogradsk District	54.917200°, 20.173978°	28.07.2023	*I. ricinus*	4
Dunaevka (Lopsienen)	Zelenogradsk District	54.867611°, 20.165678°	03.09.2023	*I. ricinus*	2
Filippovka (Dommelkeim)	Pravdinsky District	54.474473°, 20.841418°	22.05.2023	*I. ricinus*	3
				D. reticulatus	7
